# Characterisation of LV myocardial exercise function by 2-D strain deformation imaging in elite adolescent footballers

**DOI:** 10.1007/s00421-020-04510-6

**Published:** 2020-10-08

**Authors:** Guido E. Pieles, Lucy Gowing, Diane Ryding, Dave Perry, Steven R. McNally, A. Graham Stuart, Craig A. Williams

**Affiliations:** 1 Institute of Sport Exercise and Health (ISEH), University College London, London, W1T 7HA UK; 2 Bristol Congenital Heart Centre, The Bristol Heart Institute, University Hospitals Bristol NHS Foundation Trust, Upper Maudlin Street, Bristol, BS2 8BJ UK; 3 National Institute for Health Research (NIHR) Cardiovascular Biomedical Research Centre, Bristol Heart Institute, Upper Maudlin Street, Bristol, BS2 8BJ UK; 4 Manchester United Football Club, Football Medicine and Science Department, AON Training Complex, Birch Road, Carrington, Manchester, M31 4BH UK

**Keywords:** Exercise stress echocardiography, Ventricular function, Myocardial reserve, Training, Adolescent athletes

## Abstract

**Purpose:**

Few data exist on the descriptions of LV myocardial mechanics and reserve during dynamic exercise of adolescent athletes. The aim of this study was to describe the LV myocardial and cardiopulmonary changes during exercise using 2-D strain deformation imaging.

**Methods:**

Elite adolescent male football players (*n* = 42) completed simultaneous cardiopulmonary exercise testing (CPET) and exercise echocardiography measurement of LV myocardial deformation by 2-D strain imaging. LV longitudinal and circumferential 2-D strain and strain rates were analyzed at each stage during incremental exercise to a work rate of 150 W. Additionally, exercise LV myocardial deformation and its relation to metabolic exercise parameters were evaluated at each exercise stage and in recovery using repeated measures ANOVA, linear regression and paired *t* tests.

**Results:**

LV peak systolic baseline 2-D strain (longitudinal: − 15.4 ± 2.5%, circumferential: − 22.5 ± 3.1%) increased with each exercise stage, but longitudinal strain plateaued at 50 W (mean strain reserve − 7.8 ± 3.0) and did not significantly increase compared to subsequent exercise stages (*P* > 0.05), whilst circumferential strain (mean strain reserve − 11.6 ± 3.3) significantly increased (*P* < 0.05) throughout exercise up to 150 W as the dominant mechanism of exercise LV contractility increase. Regression analyses showed LV myocardial strain increased linearly relative to HR, VO_2_ and O_2_ pulse (*P* < 0.05) for circumferential deformation, but showed attenuation for longitudinal deformation.

**Conclusion:**

This study describes LV myocardial deformation dynamics by 2-D strain and provides reference values for LV myocardial strain and strain rate during exercise in adolescent footballers. It found important differences between LV longitudinal and circumferential myocardial mechanics during exercise and introduces a methodology that can be used to quantify LV function and cardiac reserve during exercise in adolescent athletes.

## Introduction

The adaptation of left ventricular (LV) morphology to athletic training in adults has been well described in several seminal publications and meta-analyses (Morganroth et al. 1975; Nishimura et al. 1980; Maron 1986; Utomi et al. 2013). But few data exist on the adaptation of the LV in the rapidly increasing population of adolescent athletes. Where available, studies in adolescent athletes have concentrated on LV morphology and cardiac functional adaptations at rest (Sharma et al. 2002; Makan et al. 2005; Di Paolo et al. 2012; Pela et al. 2016, McClean et al. 2017). Like adult athletes, adolescent athletes are also at risk of sudden cardiac death (SCD) (Malhotra et al. 2018). Importantly, 33–56% of SCD events in young athletes occur with exertion (Roberts et al. 1980; Epstein et al. 1986; Harmon et al. 2011; Chandra et al. 2013), but the underlying pathophysiological mechanisms are poorly understood, in part, because imaging data describing LV physiology during exercise in particular in adolescents are still rare. Exercise stress echocardiography has recently, however, been shown to differentiate physiological LV functional adaptive processes from myocardial disease, where function decreases, in adult athletes (La Gerche et al. 2013; Sanz-de la Garza et al. 2017) and it has also been shown to unmask cardiac dysfunction that is not detectable at rest in the paediatric and adolescent congenital heart disease population (Roche et al. 2011, Roche et al. 2014). No studies have so far described LV myocardial response during exercise in adolescent athletes. The gold standard methodology for LV myocardial performance assessment, also during exercise, is contractility assessment by end-systolic elastance using conductance catheters, which is an invasive technique (Izawa et al. 1996; Inagaki et al. 1999). To overcome this problem and particularly important in the paediatric population, myocardial deformation imaging by 2-D strain during exercise has recently been shown to present a more practical alternative. Furthermore, proof of principle studies including reference values for 2-D strain during exercise in non-athlete adolescents have recently become available (Boissiere et al. 2013; Pieles et al. 2015; Cifra et al. 2016).

2D strain imaging, including measurement of peak systolic strain and strain rate, is less load-dependent than other classic echocardiographic techniques of LV function, such as ejection fraction (Weidemann et al. 2002a, b). These considerations are paramount when assessing LV function during exercise with its significant pre- and after-load changes, additionally 2-D strain imaging shows angle independency, which is important to counteract significant translational heart movement during exercise. Importantly, 2-D strain at rest has been shown to differentiate adaptive from maladaptive processes in adult athletes (Kansal et al. 2011). The application of 2-D strain imaging during exercise stress echocardiography is, therefore, an appropriate methodology to enhance current practice in quantitatively assessing myocardial function and reserve in adolescent athletes.

One further challenge that remains is to integrate cardiac exercise function to the measurement of other organ systems. Cardio-pulmonary exercise testing (CPET) is the gold standard (Astrand 1971; Paridon et al. 2006) and has been used in diagnosis, risk stratification and outcome prediction in children and adults with cardiac disease (Rhodes et al. 2010; Guazzi et al. 2012). However, a major limitation of CPET is that it does not provide direct data on exercise-related changes in myocardial function or cardiac reserve (Bassett and Howley 2000). Simultaneous measurement of cardiac performance by 2-D strain echocardiography and metabolic exercise response by CPET can, thus, overcome this limitation and a pilot study from our group has shown its suitability in healthy adolescent volunteers (Pieles et al. 2015). Therefore, the aim of this study was to utilise the integrated methodology of exercise echocardiography with CPET to describe LV myocardial exercise response in relation to exercise metabolism during strenuous exercise in adolescent elite footballers.

## Methods

### Participants

Forty-two healthy elite male players from an English Premier League football academy (mean age 15.4 ± 1.7 y, stature 172.2 ± 9.7 cm, body mass 58.7 ± 11.0 kg, BMI 19.6 ± 2.1 kg m^2^, lean body mass 47.2 ± 7.5 kg, body surface area 1.69 ± 0.20 m^2^), volunteered to participate in this prospective cohort study and prior to participation, parent/carer and adolescents duly signed a consent form and/or an assent form, respectively. UK National Research Ethics Service (NRES) approval was obtained. Participants were screened for cardiac disease by pre-participation questionnaire physical examination, 12-lead ECG and resting echocardiography. National elite training status was defined as consisting of a minimum of 12 h per week training and game time and selection, actively participating in competition including international tournaments and possessing a contract into the clubs elite training programme (Araujo and Scharhag 2016).

### Cardio-pulmonary exercise testing

An incremental CPET on a recumbent cycle ergometer (Ergosana GMBH, Bitz, Germany) positioned at a 45° inclination (25 W∙3 min^−1^ increments) was performed to volitional exhaustion at a pedaling frequency of 70 ± 5 rpm. Exercise stages of 3 min were used to obtain a steady state and enough time to obtain echocardiographic data. Ventilation volume and expired gas composition were measured breath-by-breath using a metabolic cart (Metalyzer II, Cortex, Leipzig, Germany) with calibration, measurement and analysis of metabolic gas parameters during exercise and recovery performed as described previously (Pieles et al. 2015). Participants were requested to avoid strenuous exercise for at least 12 h preceding each visit and to arrive at the laboratory in a rested and hydrated state 2 h after a meal.

### Echocardiography

Prior to exercise stress testing, participants underwent a full structural and functional resting (baseline) echocardiogram following international paediatric guidelines (Lai et al. 2006, Lopez et al. 2010). Echocardiographic measurements and analysis were performed using an Artida machine and a 2.0–4.8 MHz transducer and UltraExtendV3.2 software (Canon Medical Systems, Japan). LV diameters were measured from two-dimensional (2-D) echocardiography in the parasternal short axis view at the base of the LV. Ejection fraction (EF) was calculated using the Simpson 2-D biplane method.

### 2-D myocardial strain analysis

A parasternal short axis and LV focused apical 4-chamber view were captured for 2-D strain analysis. Three cardiac cycles were acquired at rates of 60–100 frames per second (Fps), analysis was performed on one manually selected cardiac cycle. The endocardial borders were manually contoured at end-systole with the range of interest adjusted to include the whole myocardium. Mean peak systolic longitudinal (Sl) and circumferential (Sc) strain were defined as the maximal deformation value of a segment during systole in the endocardial segment and is represented as a percentage (%); mean peak systolic strain rate (SR) was defined as the maximal rate of deformation of a segment in systole over time and is expressed in 1/s (Voigt et al. 2015). Circumferential peak systolic strain was measured at the base of the LV. Mean values for circumferential and longitudinal strain were calculated for each stage only if good tracking was obtained in a minimum of four segments. Image acquisition and off-line analysis were performed by an investigator experienced in paediatric echocardiography (GEP).

### Exercise stress echocardiography

**Fig. 1:**
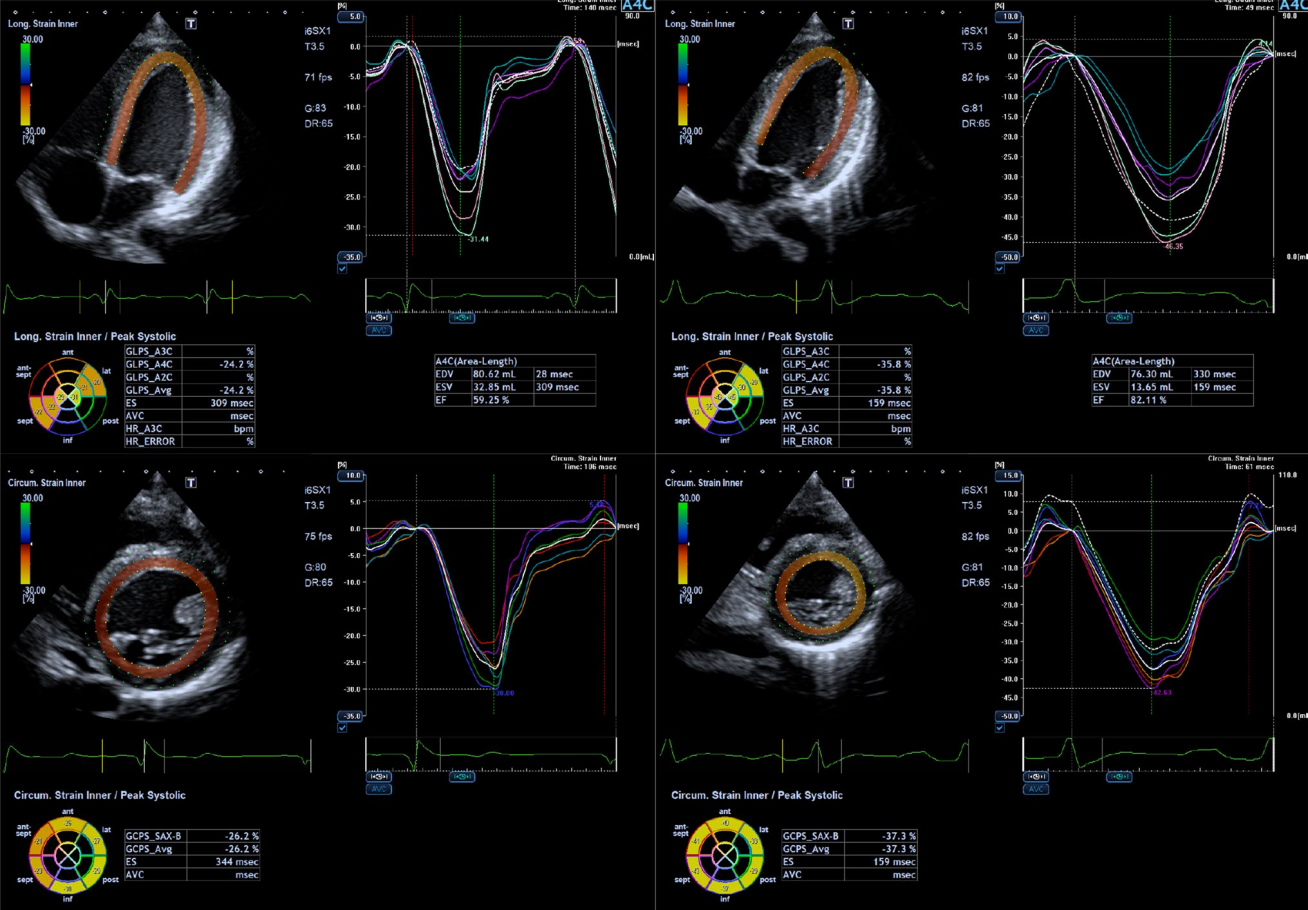
Representative 2-D strain images and strain curves for LV Sl (top) and LV Sc (bottom) at baseline (left, HR = 72 bpm) and during moderate exercise (right, 100 W, HR = 134 bpm)

Exercise stress echocardiography and 2-D strain analysis were performed using the same protocol and by the same internationally accredited operator, as described by our research group previously (Pieles et al. 2015). Briefly, focused echocardiography was performed for 2-D strain analysis during free breathing exercise 60 s into each exercise stage at baseline (rest), 0 (unloaded pedaling), 50, 100, 150 W and during recovery at 2 min (Rec2) and 6 min (Rec6) after end of exercise. The gas exchange threshold (GET), representing the break point in breath-by-breath values of carbon dioxide uptake and oxygen uptake was expressed as a percentage of VO_2peak_. Myocardial reserve was defined as the difference in 2-D mean peak systolic strain between baseline and each exercise stage to up to 150 W. Strain values were not calculated at work rates higher than 150 W to ensure sufficient image quality and frame rate for reliable strain analysis. Only images with high frame rates of 60–100 Fps were used to ensure capture of sufficient Fps for 2-D strain analysis at higher heart rates. A minimum of 3 cardiac cycles were recorded to capture at least one cardiac cycle in expiration to obtain best image quality, which was confirmed visually and then used to perform strain analysis (Fig. 1).

### Statistics

**Fig. 2:**
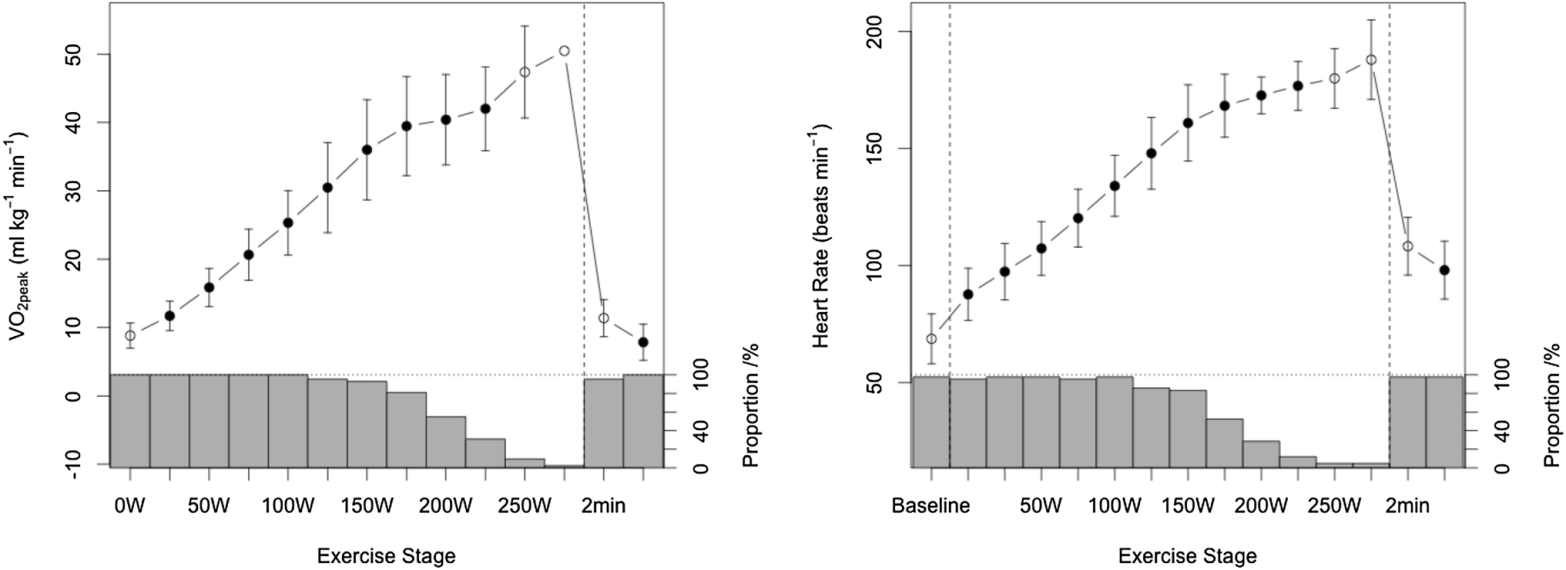
Mean (SD) responses of VO_2_ (left) and HR (right) up to peak exercise and during recovery for participants. Proportion of participants that reached the respective work rates are indicated. Filled dots show statistical difference compared to the subsequent stage at the *P* < 0.05 level. Please see also Table 1

**Fig. 3:**
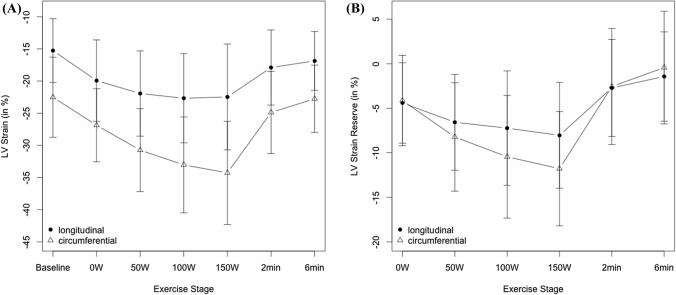
**a** Mean LV peak systolic longitudinal strain and circumferential strain at each exercise stage (baseline to 6 min recovery); **b** Mean LV peak systolic longitudinal strain and circumferential strain reserve at each exercise stage (baseline to 6 min recovery) showing a plateauing effect for mean peak systolic LV longitudinal strain

**Fig. 4:**
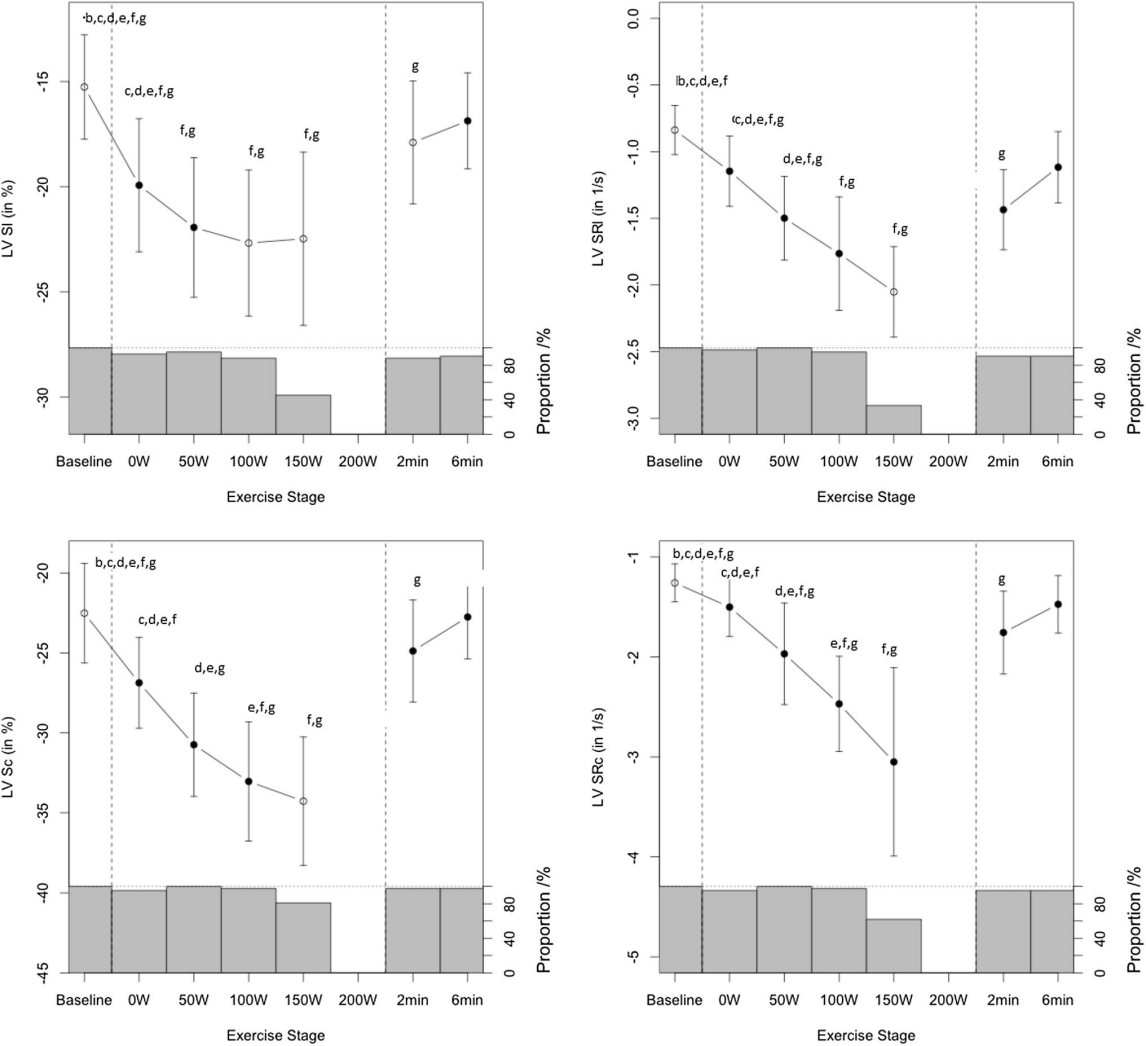
Myocardial performance response during exercise as measured by mean LV peak systolic longitudinal (Sl) and circumferential (Sc) strain and strain rate (SRl, SRc), respectively. Letters above each stage show statistical significant difference (ANOVA and post-hoc test) in comparison with the selected exercise stage, such that letters correspond to: b = 0 W, c = 50 W, d = 100 W, e = 150 W, f = 2 min rec and g = 6 min rec; (e.g. in left upper panel at baseline significant differences found compared to 0, 50, 100, 150, 2 min rec and 6 min rec)

**Fig. 5:**
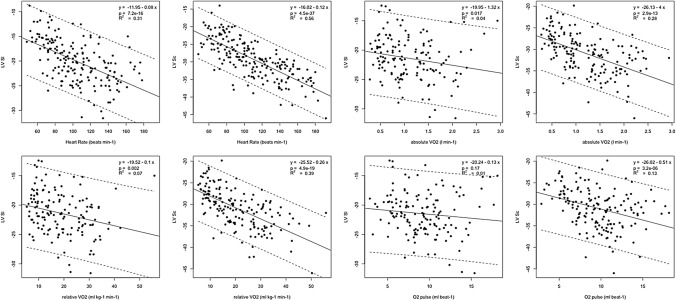
Scatterplots representing the increase of LV myocardial performance as measured by mean LV peak systolic longitudinal (Sl) and circumferential (Sc) strain in relation to HR and metabolic exercise parameters

**Fig. 6:**
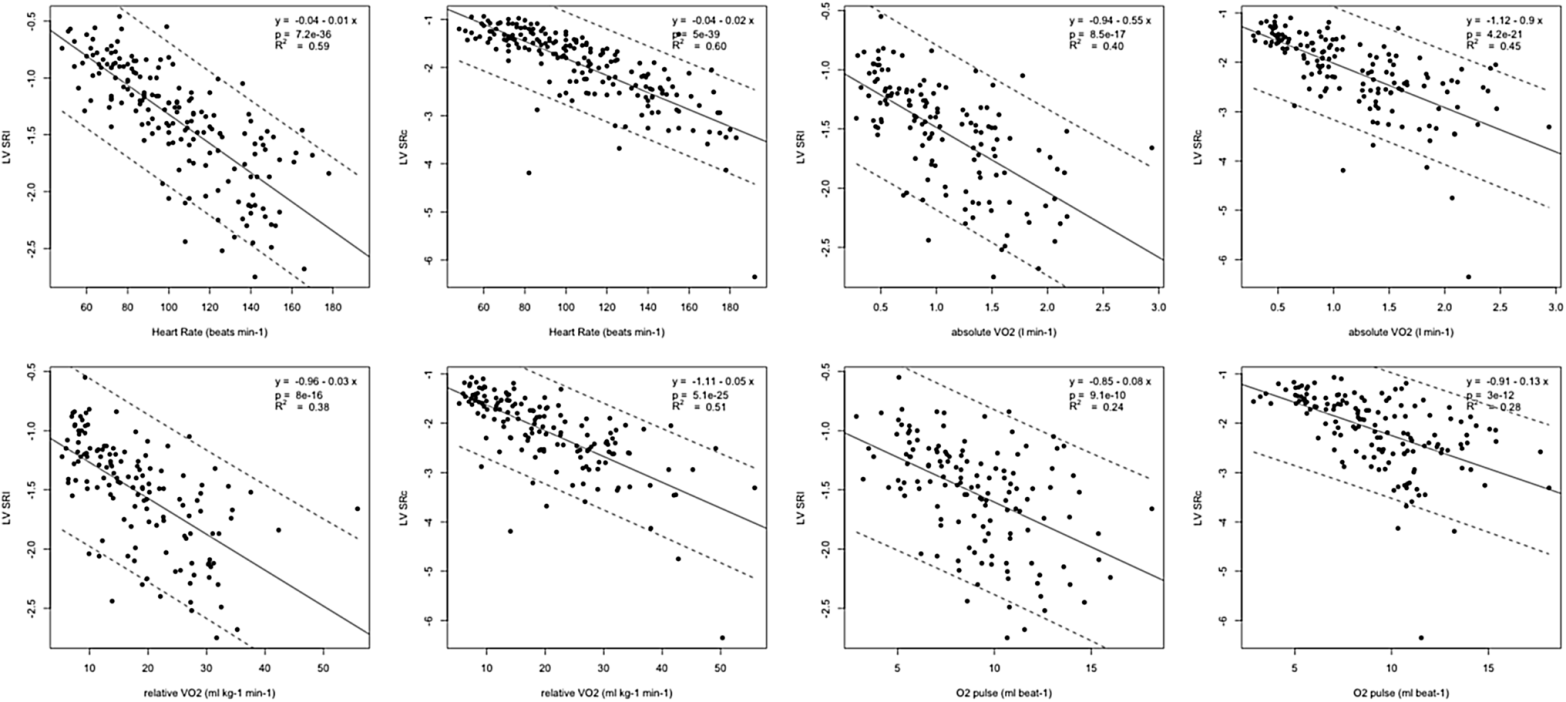
Scatterplots representing the increase of LV myocardial performance as measured by mean LV peak systolic longitudinal (SRl) and circumferential (SRc) strain rate in relation to HR and metabolic exercise parameters

Descriptive statistics (mean and SD) of measured and derived variables were used to characterize the sample (Table 1). Prior to analyses, diagnostic plots were created to provide checks for heteroscedasticity, normality and influential observations (Figs. 2, 3). Outliers were identified as being > 1.5 times the IQR (interquartile range) using box and whisker plots. Differences between strain and strain rate and were assessed with repeated measures ANOVA and Least Significance Difference post hoc test. Strain reserve variables were assessed using paired *t *tests to compare between rest and individual exercise stage only. Development of myocardial deformation was tested and is described as the difference between each subsequent exercise stage (Fig. 4), as well as the differences compared to rest (Tables 2 and 3). Relationships between strain parameters and CPET variables were determined using scatterplots, Pearson’s correlations and linear regression analysis (Figs. 5 and 6). All statistical analyses were performed using R Core Team (Vienna, Austria) and using SPSS Statistics Software (Version20.0. IBM Corp, USA) and GraphPad Prism (Version5.04, La Jolla, USA). A probability level of *P* < 0.05 was accepted to indicate statistical significance.

**Table 1:** Baseline, exercise, and recovery cardiopulmonary exercise testing parameters of the athlete cohort (*n* = 42)

Test stage	Absolute VO_2_, L∙min^−1^	Relative VO_2_, mL∙kg^−1^∙min^−1^	Heart rate, b∙min^−1^	Oxygen pulse, mL∙beat^−1^	VO_2_/VO_2peak_ %	Participants (*n*)
Baseline	–	–	69 ± 11	–		42
0 W	0.51 ± 0.10	8.86 ± 1.84	88 ± 11	5.90 ± 1.51	18.0 ± 3.2	42
25 W	0.67 ± 0.12	11.71 ± 2.15	97 ± 12	7.05 ± 1.70	24.0 ± 4.7	42
50 W	0.91 ± 0.10	15.86 ± 2.78	107 ± 11	8.55 ± 1.51	32.5 ± 6	42
75 W	1.18 ± 0.12	20.64 ± 3.74	120 ± 12	9.91 ± 1.59	42.4 ± 8.6	42
100 W	1.44 ± 0.13	25.33 ± 4.71	134 ± 13	10.84 ± 1.50	51.9 ± 10	42
125 W	1.75 ± 0.18	30.46 ± 6.59	148 ± 15	11.89 ± 1.65	61.4 ± 11.5	40
150 W	2.10 ± 0.26	36.00 ± 7.32	161 ± 16	13.30 ± 2.26	72.5 ± 12	39
175 W	2.34 ± 0.23	39.46 ± 7.24	168 ± 13	14.02 ± 1.89	78.5 ± 11.0	34
200 W	2.55 ± 0.17	40.40 ± 6.61	173 ± 8	15.02 ± 1.39	81.8 ± 9.2	23
225 W	2.91 ± 0.26	42.00 ± 6.13	177 ± 10	17.58 ± 1.20	85.5 ± 10.2	13
250 W	3.47 ± 0.51	47.37 ± 6.73	180 ± 13	20.54 ± 0.18	88.5 ± 8.5	4
275 W	3.90 ± N/A	50.47 ± N/A	200 ± N/A	19.48 ± N/A	83.4 ± N/A	1
Max exercise	2.46 ± 0.62	49.11 ± 6.54	173 ± 13	13.29 ± 3.04	87.2 ± 7.1	42
2 min rec	0.66 ± 0.23	11.25 ± 2.81	109 ± 13	6.09 ± 1.86	23.2 ± 5.3	42
6 min rec	0.47 ± 0.21	7.85 ± 2.64	98 ± 12	4.86 ± 1.89	16.1 ± 5.8	42

CI is defined as the mean ± 2 standard deviations

**Table 2:** Mean LV peak systolic longitudinal and LV peak systolic circumferential strain response during exercise

Test stage	Strain value	SD	Lower CI	Upper CI	Strain reserve	*P* value	Participants (*n*)
LV peak systolic longitudinal strain							
Baseline	− 15.3	2.5	− 20.2	− 10.3	0 ± 0	–	42
0 W	− 19.9	3.2	− 26.3	− 13.6	− 4.4 ± 2.3	< 0.001	39
50 W	− 21.9	3.3	− 28.6	− 15.3	− 6.6 ± 2.7	< 0.001	40
100 W	− 22.7	3.5	− 29.6	− 15.7	− 7.2 ± 3.2	< 0.001	37
150 W	− 22.5	4.1	− 30.7	− 14.2	− 8 ± 3	< 0.001	19
2 min rec	− 17.9	2.9	− 23.7	− 12.1	− 2.7 ± 2.7	< 0.001	37
6 min rec	− 16.9	2.3	− 21.4	− 12.3	− 1.4 ± 2.5	0.003	38
LV peak systolic circumferential strain							
Baseline	− 22.5	3.1	− 28.7	− 16.3	0 ± 0	< 0.001	42
0 W	− 26.9	2.8	− 32.6	− 21.2	− 4.1 ± 2.5	< 0.001	40
50 W	− 30.7	3.2	− 37.2	− 24.3	− 8.2 ± 3	< 0.001	42
100 W	− 33.0	3.7	− 40.5	− 25.6	− 10.5 ± 3.4	< 0.001	41
150 W	− 34.3	4.0	− 42.3	− 26.2	− 11.8 ± 3.2	< 0.001	34
2 min rec	− 24.9	3.2	− 31.3	− 18.5	− 2.6 ± 3.3	< 0.001	41
6 min rec	− 22.8	2.6	− 28.0	− 17.5	− 0.4 ± 3.2	< 0.001	41

*P* values compare strain parameters at each exercise stage compared to baseline. CI is defined as the mean ± 2 standard deviations

**Table 3:** Mean LV peak systolic longitudinal and LV peak systolic circumferential strain rate response during exercise

Test stage	Strain rate value	SD	Lower CI	Upper CI	SR reserve	*P* value	Participants (*n*)
LV peak systolic longitudinal strain rate							
Baseline	− 0.84	0.18	− 1.21	− 0.47	0 ± 0	–	42
0 W	− 1.15	0.26	− 1.67	− 0.62	− 0.3 ± 0.3	< 0.001	41
50 W	− 1.50	0.31	− 2.13	− 0.87	− 0.7 ± 0.3	< 0.001	42
100 W	− 1.76	0.43	− 2.62	− 0.91	− 0.9 ± 0.5	< 0.001	40
150 W	− 2.05	0.34	− 2.73	− 1.37	− 1.3 ± 0.4	< 0.001	14
2 min rec	− 1.44	0.30	− 2.04	− 0.84	− 0.6 ± 0.3	< 0.001	38
6 min rec	− 1.12	0.27	− 1.65	− 0.58	− 0.3 ± 0.3	< 0.001	38
LV peak systolic circumferential strain rate							
Baseline	− 1.26	0.19	− 1.64	− 0.88	0 ± 0	–	42
0 W	− 1.50	0.29	− 2.09	− 0.91	− 0.2 ± 0.3	< 0.001	40
50 W	− 1.97	0.51	− 2.98	− 0.95	− 0.7 ± 0.6	< 0.001	42
100 W	− 2.47	0.48	− 3.42	− 1.52	− 1.2 ± 0.5	< 0.001	41
150 W	− 3.05	0.94	− 4.93	− 1.17	− 1.8 ± 1	< 0.001	26
2 min	− 1.76	0.41	− 2.58	− 0.93	− 0.5 ± 0.4	< 0.001	40
6 min	− 1.47	0.29	− 2.05	− 0.90	− 0.2 ± 0.3	< 0.001	40

*P* values compare strain parameters at each exercise stage compared to baseline. CI is defined as the mean ± 2 standard deviations

## Results

All participants had a structurally and functionally normal heart. Participants had normal LV dimensions (IVSd 9.7 ± 1.3 mm, LVPWd 8.9 ± 1.3 mm, LVIDd 44.5 ± 3.8 mm, LVIDs 32 ± 5.8 mm) with all individual z scores within ± 2 and normal LV systolic function with a mean FS of 33 ± 3% and a mean EF of 64 ± 5%. Table 1 represents the mean (SD) CPET data. The exercise duration was 25:44 ± 5:46 (min:s) with a mean peak power output of 211 ± 45 W. Relative VO_2peak_ was 49.1 ± 6.5 mL·kg^−1^·min^−1^, GET was 129 ± 38 W and 69 ± 13% of VO_2peak_ or 33.3 mL·kg^−1^·min^−1^. Oxygen consumption and HR as shown in Fig. 2 significantly increased linearly up to end exercise (*P* < 0.05).

### LV myocardial performance during exercise—2-D strain

Analysis of 2-D LV strain was feasible up to a work rate of 100 W and a mean HR of 134 ± 13 b∙min^−1^ in 90% of subjects for longitudinal and 98% of subjects for circumferential strain and to 150 W and a mean HR of 161 ± 16 b∙min^−1^ in 60% of subjects for longitudinal and 88% for circumferential 2-D strain (Table 2). Initiation of exercise (0 W) resulted in a significant increase of LV mean peak systolic longitudinal strain (LV Sl) compared to baseline (*P* = 0.001) and this linear increase was maintained up to 50 W (*P* = 0.001). A plateauing effect at the higher power outputs between 50 and 150 W was shown (Fig. 4), where inter-stage comparisons were not significantly different between 50 and 100 W (*P* = 0.06) and 100 and 150 W (*P* = 0.91) (Fig. 4.). This plateau effect for LV Sl corresponded to a VO_2peak_ of between 52 and 75% (Table 1 and Figs. 3 and 4) falling into the range, where GET occurred, specifically 12% and 59% of participants were at GET at 100 W and 150 W, respectively. In contrast, inter-stage comparisons from baseline and exercise stages up to 150 W for LV mean peak systolic circumferential strain (Sc) showed a linear and significant increase (all comparisons *P* = 0.001) (Fig. 4). LV Sl and LV Sc showed a similar profile during the recovery, during immediate recovery (2 min) period decreasing towards baseline level to 120% (Sl) and 114% (Sc) of baseline strain, respectively, and continuing this trend to 6 min recovery. Importantly however, values did not reach baseline values at 6 min recovery (*P* < 0.05) (Table 2 and 3). Myocardial reserve measured by 2-D strain was more pronounced in the circumferential plane, LV Sl reserve was − 7.8 ± 3.0 vs − 11.6 ± 3.3 for LV Sc reserve (Fig. 3). Strain rate (SR) during exercise increased significantly (*P* = 0.001) between most exercise stages for the longitudinal and circumferential myofibre direction and did not reach a plateau at higher exercise stages (Table 3 and Fig. 4). Three comparisons for longitudinal SR between 0 W and 6 min rec (*P* = 0.84) and 50 W and 2 min rec (*P* = 0.65) and circumferential SR for 0 W and 6 min rec (*P* = 0.75) were found not to be significantly different. Strain rate reserve was significantly different at all stages compared to baseline (*P* < 0.001) (Table 3).

### Force–Frequency relationship

Figures 5 and 6 represent the scatterplot of the relationship between strain, strain rate and chronotropic exercise response. Both, strain and strain rate increased with HR during exercise in a moderate linear relationship, confirming a positive physiological force–frequency relationship (FFR) between HR and all investigated strain and strain rate parameters (*P* < 0.001). Sc showed a stronger correlation to heart rate than Sl (*R*^2^ = 0.56 vs 0.31, *P* < 0.001) (Fig. 5). Compared to strain, strain rate showed a stronger relationship for all strain rate values (Figs. 5 and 6).

### Relationship between exercise myocardial performance and metabolic exercise parameters

LV Sl, LV Sc, LV SRl and LV SRc increased linearly to absolute and relative VO_2_ and O_2_ pulse, the positive relationships are shown in the scatterplots in Figs. 5 and 6. The strength of the linear relationships was stronger for strain rate compared to strain and was also stronger for LV Sc compared to LV Sl for all variables assessed (Figs. 5 and 6).

## Discussion

In this study, we describe for the first time, the LV myocardial contractile response to exercise in elite adolescent footballers by 2-D strain imaging. Using a novel approach piloted previously by our group (Pieles et al. 2015), which combines 2-D strain imaging with simultaneous metabolic evaluation using CPET, we investigated the mechanics of LV contraction under exercise stress, as well as the relationship of exercise LV function to metabolic exercise parameters.

### LV myocardial performance during exercise

Strain and strain rates at baseline were comparable to published reference data in healthy adolescents (Marcus et al. 2011). We found an incremental increase in LV Sl and LV Sc in response to exercise and in accordance to previous exercise strain assessment studies in adult elite athletes (La Gerche et al. 2012). The increase in LV Sc, a parameter of myocardial contractility of circumferential myocardiac fibres, was more pronounced and increased more linearly to 150 W whereas LV Sl, which describes contractility of longitudinal myocardial fibres, reached a plateau at moderate work rates (50–100 W), coinciding with GET as a possible associated mechanism. The contribution of circumferential myocardial contractility was more pronounced as shown by a higher absolute strain increase for circumferential strain over longitudinal strain (− 34.0 ± 4% vs − 22.4 ± 4%), as well as a higher circumferential strain reserve (− 11.6 ± 3.3 vs − 7.8 ± 3.0) (Figs. 3 and 4). This observation points towards a differential exercise contribution of LV longitudinal and circumferential myocardial fibres with dominance of circumferential myofibre motion at higher work rates. This is in accordance with one previous adult study (Stohr et al. 2014) and also confirmed by animal studies (Kovacs et al. 2015), that showed recruitment of circumferential myofibres during exercise confers a higher contractility. This observation is also in accordance with data from non-athlete adolescents in our previous study (Pieles et al. 2015). While specific loading conditions during exercise might to some degree influence our 2-D strain values (Greenberg et al. 2002), we validated this result by measuring strain rate (Table 3 and Fig. 6), which is the least load-dependent parameter (Ferferieva et al. 2012), and in accordance, absolute circumferential systolic strain rate increase was also more pronounced than longitudinal systolic strain (− 3.71 ± 0.71 vs − 2.05 ± 0.34) as was circumferential strain rate reserve (− 2.51 ± 0.77 vs − 1.29 ± 0.37). In contrast to strain, however, strain rate increased continuously to 150 W without a plateau in both fibre directions. Strain rate has previously been shown to be the most accurate noninvasive measurement of contractility during dobutamine (Greenberg et al. 2002) and exercise stress (La Gerche et al. 2012) also compared to strain. As a derivative of myocardial velocities, it is very sensitive and less influenced by pre- and afterload changes and translational tissue motion changes that even strain is susceptible to (Greenberg et al. 2002). While intrinsic myofiber contractile force still increases at higher exercise stages leading to continuous strain rate increase, this phenomenon would not have been captured as well by strain. The plateauing effect of strain values in our study, can be hypothesized to be a result of the susceptibility of strain to stroke volume changes (Weidemann et al. 2002a, b) which has been shown to not alter significantly at near maximal exercise (Higginbotham et al. 1986). Our previous data in healthy non-athlete adolescents of similar age using the same methodology (Pieles et al. 2015) showed similar development for strain and strain rate.

### Force–Frequency relationship

Both, strain and strain rate followed the physiological force–frequency relationship (FFR), a fundamental relationship of healthy myocardium demonstrated in the paediatric population (Roche et al. 2011), by an incremental increase in contractility as measured by strain and strain rate in relation to HR. An abnormal FFR during exercise has recently been shown in children with heart disease (Roche et al. 2014), and hence the data on FFR development during exercise presented here could become a tool to detect early LV dysfunction in adolescent athletes. For athletes in particular, the detailed description of LV myocardial performance during exercise is of importance, as reduced 2-D strain and exercise-induced LV dysfunction are early signs in adolescent and adult subclinical hypertrophic cardiomyopathy (Sakata et al. 2008; Forsey et al. 2014), which is a major cause of sudden cardiac death in the athlete population (Malhotra et al. 2018). Hence, the use of 2-D strain during exercise stress echocardiography has the potential to further increase the utility of 2-D strain in the early detection of myocardial disease, in particular in athletes where myocardial disease is often concealed and remains a diagnostic challenge (Sheikh et al. 2015).

### LV myocardial performance in recovery

2-D strain values at recovery showed a tendency to return to baseline values, but showed significant differences between baseline and recovery (Fig. 4 and Table 2). Equally, strain rate, as the best measure of contractility, did not return to baseline at 2 and 6 min recovery (Table 3), both strain and strain rate recovery mechanics indicating a higher myocardial performance demand in the recovery phase. This will also have been influenced by the higher HR via the described FFR compared to pre-exercise baseline values (Table 1). We previously described albeit statistically non-significant, a reduction of 2-D strain during recovery in a non-athlete adolescent cohort as a reflection of altered myocardial recovery function after maximal exercise (Pieles et al. 2015), but this was not observed in the athlete cohort, which might present a specific athletic myocardial adaptation, but would require a direct comparison study, that was beyond the scope of this paper.

### Myocardial performance and metabolic exercise parameters

Study participants showed higher fitness levels with a superior VO_2peak_ compared to previous published data in age matched non-athlete adolescents using the same exercise test methodology (Pieles et al. 2015). Ventricular mechanics are highly dependent not only on HR as discussed above, but also on exercise intensity, work rate and metabolic state (Armstrong et al. 2016). We have, therefore, also investigated the relationships between longitudinal and circumferential myocardial performance by strain and strain rate and absolute and relative VO_2_ and O_2_ pulse. Scatterplots confirmed a linear relationship (Figs. 5 and 6) for absolute and relative VO_2_ and O_2_ pulse. Importantly, as *R*^2^ values (Figs. 5 and 6) show, there exists a stronger relationship of absolute and relative VO_2_ and O_2_ pulse with Sc compared to Sl also indicating a dominance of circumferential fibre shortening during exercise as discussed above. The relationship between strain and O_2_ pulse found supports the clinical use of peak O_2_ pulse as a surrogate parameter for cardiac function during clinical CPET testing, and vice versa, validating 2-D strain during exercise as an alternative tool to more accurately and directly measure LV myocardial function during clinical exercise testing. Here, Sc might be the superior parameter over Sl to be used during exercise stress echocardiography judging by its closer relationship to absolute and relative VO_2_ and O_2_ pulse. Further work is needed to determine if this relationship is different in the setting of myocardial disease.

## Limitations

There are a number of technical challenges when assessing simultaneous echocardiography and CPET that need to be acknowledged. First is the requirement for a semi-supine position to acquire satisfactory images resulting in a position-specific CPET response (Warburton et al. 2002), which was shown to be feasible and valid in children (May et al. 2013). Second, as a result of insufficient HR vs frame rate ratio, 2-D strain measurements were not performed at the maximal end-exercise stage of the test. Tissue Doppler imaging with higher acquisition frame rates could theoretically overcome this, however, this technique is angle-dependent and paediatric exercise studies (Cifra et al. 2016) have not yielded data at maximal exercise intensities. Good intra- and inter-observer reliability of 2-D strain measurements, paramount during exercise echocardiography (Picano et al. 1991), using the same hard- and software, protocol and the same echocardiographer were established in our previous pilot study (Pieles et al. 2015). The intra- and interobserver average variance for baseline, exercise, and recovery strain values there ranged from 0.6 to 8% with and intraclass correlation coefficient (ICC) between *r* = 0.78 and 0.98 for LV Sl; from 0.9 to 9.1% with ICC between *r* = 0.87 and 0.98 for LV Sc. Our study cohort was extremely homogenous and a more heterogeneous sample might have provided more subtle inter-subject associations. While this is, to our knowledge to date, the largest cohort study using 2-D strain exercise echocardiography in adolescent athletes, further larger studies will need to be conducted to create large-scale normative data.

## Conclusion

This study characterized the LV myocardial mechanics during exercise in elite adolescent athletes using 2-D strain imaging. It describes the normal response of myocardial function during exercise and recovery and showed, that there is a specific response of longitudinal and circumferential myocardial performance to exercise stress, knowledge that in the future might help differentiate between adaptive and maladaptive myocardial function in paediatric athletes and those with myocardial disease. Additionally, it provides the first initial reference data for 2-D strain and strain rate values of the LV during exercise in the healthy adolescent elite athlete population.

## Abbreviations

CPET: Cardio-pulmonary exercise testing
EF: Ejection faction
FFR: Force–frequency relationship
Fps: Frames per second
FS: Fractional shortening
GET: Gas exchange threshold
IVSd: Interventricular septal diastolic diameter
LV: Left ventricle
LVIDd: Left ventricular internal diastolic diameter
LVIDs: Left ventricular internal systolic diameter
LVPWd: Left ventricular posterior wall diastolic diameter
Sc: Circumferential peak systolic strain
SCD: Sudden cardiac death
Sl: Longitudinal peak systolic strain
SRc: Circumferential peak systolic strain rate
SRl: Longitudinal peak systolic strain rate
VO_2peak_: Peak oxygen consumption
W: Watts
2-D strain: 2-Dimensional strain
